# PReS-FINAL-2361: Maintenance treatment in childhood granulomatosis with polyangiitis

**DOI:** 10.1186/1546-0096-11-S2-P351

**Published:** 2013-12-05

**Authors:** M Twilt, R Schneider, D hebert, E harvey, R laxer, S dell, C licht, S benseler

**Affiliations:** 1 Rheumatology, Birmingham children's Hospital, Birmingham, UK; 2 Rheumatology, the hospital for sick children, Toronto, Canada; 3 Nephrology, the hospital for sick children, Toronto, Canada; 4 Respiratory medicine, the hospital for sick children, Toronto, Canada

## Introduction

Granulomatosis with Polyangiitis (GPA) is a rare but life threatening disease. Most children present with pulmonary bleeds and/or renal failure. Most treatment regimens are derived from the adult literature, no studies have been performed in pediatric patients.

## Objectives

The aim of this study is to describe the maintenance treatment in a large group of children with GPA.

## Methods

All consecutive children diagnosed with GPA since January 2000 in the Hospital for Sick Children were included. Demographic data, data at diagnosis and follow-up data were collected. Descriptive statistics were used for these preliminary results.

## Results

32 children were diagnosed with GPA since January 2000. Twenty-one girls and 11 boys, with a median age of 13.7 years at diagnosis. ANCA was positive in 30 children (26 c-ANCA with 25 anti-PR-3, 4 p-ANCA with 4 anti MPO) and 2 were ANCA negative (1 anti-PR3 positive). Eight children had limited disease and 24 systemic disease. All systemic patients were treated with pulses cyclophospamide iv (mean 7 pulses) and methylprednisone (mean 5 pulses) iv, and 6 children received plasmapheresis. Maintenance treatment in this group consisted of MTX in 7, AZA in 14, MMF in 3 children. In the limited disease group, treatment consited of oral prednisone in all, MTX in 7 children and AZA in 1. Relapses were seen in 14 children. One child did not receive any treatment at time of relapse. Two children with limited disease relapsed, both while still on MTX. 11 children with systemic disease were still on treatment, MTX in 4, AZA in 5, MMF in 2.

## Conclusion

Relapses are seen often in childhood GPA when still receiving maintenance treatment. Relapses are higher in children with systemic GPA (50%) compared to limited GPA (25%).

## Disclosure of interest

M. Twilt: None Declared, R. Schneider: None Declared, D. hebert: None Declared, E. harvey: None Declared, R. laxer Grant/Research Support from: novartis, S. dell: None Declared, C. licht: None Declared, S. benseler: None Declared.

